# Observation of Different Catalytic Activity of Various 1-Olefins during Ethylene/1-Olefin Copolymerization with Homogeneous Metallocene Catalysts 

**DOI:** 10.3390/molecules16010373

**Published:** 2011-01-07

**Authors:** Mingkwan Wannaborworn, Piyasan Praserthdam, Bunjerd Jongsomjit

**Affiliations:** Center of Excellence on Catalysis and Catalytic Reaction Engineering, Department of Chemical Engineering, Faculty of Engineering, Chulalongkorn University, Bangkok 10330, Thailand

**Keywords:** metallocene catalyst, homogeneous catalyst, long chain olefins, copolymerization

## Abstract

This research aimed to investigate the copolymerization of ethylene and various 1-olefins. The comonomer lengths were varied from 1-hexene (1-C_6_) up to 1-octadecene (1-C_18_) in order to study the effect of comonomer chain length on the activity and properties of the polymer in the metallocene/MAO catalyst system. The results indicated that two distinct cases can be described for the effect of 1-olefin chain length on the activity. Considering the short chain length comonomers, such as 1-hexene, 1-octene and 1-decene, it is obvious that the polymerization activity decreased when the length of comonomer was higher, which is probably due to increased steric hindrance at the catalytic center hindering the insertion of ethylene monomer to the active sites, hence, the polymerization rate decreased. On the contrary, for the longer chain 1-olefins, namely 1-dodecene, 1-tetradecene and 1-octadecene, an increase in the comonomer chain length resulted in better activity due to the opening of the gap aperture between C_p_(centroid)-M-C_p_-(centroid), which forced the coordination site to open more. This effect facilitated the polymerization of the ethylene monomer at the catalytic sites, and thus, the activity increased. The copolymers obtained were further characterized using thermal analysis, X-ray diffraction spectroscopy and ^13^C-NMR techniques. It could be seen that the melting temperature and comonomer distribution were not affected by the 1-olefin chain length. The polymer crystallinity decreased slightly with increasing comonomer chain length. Moreover, all the synthesized polymers were typical LLDPE having random comonomer distribution.

## 1. Introduction

Nowadays, polymers play a significant role in many applications, especially linear low-density polyethylene (LLDPE). The LLDPE has many advantages such as low density, good mechanical properties, and easy fabrication and recycling. Therefore, it has been used to produce many products such as shopping bags, food packaging film, plastic pipe and house appliances, *etc*. [1,2,3]. Thus, the demand for LLDPE is quite high compared with other polymers. For the production of LLDPE, the polymer can be synthesized by the polymerization of ethylene and short chain 1-olefins, namely 1-hexene, 1-octene and 1-decene, in the catalyst system for better activity. A low pressure slurry process, the gas phase process and the solution-phase process [4] can be employed for LLDPE. Some 15 million tons of LLDPE are produced worldwide using the metallocene catalyst system, since this catalyst can incorporate many types of comonomer. Moreover, it can give a narrow molecular weight distribution. Thus, there has been an increase in research and development on the synthesis of the LLDPE using metallocene catalysts [5,6].

However, the properties of LLDPE, such as the average molecular weight of the macromolecules and its distribution, the degree of crystallization, the melting temperature and the amount and distribution of the monomeric units, depend on a factor called “comonomer effect” [7,8,9]. Previous studies show that an increase in the quantities of 1-olefin provides higher activity which relates to a physical phenomenon improving the monomer diffusion in the lower crystalline copolymer structure. Besides the comonomer quantity, the length of the comonomer also affects the properties of LLDPE. Although short chain comonomers are normally used in the process, long chain comonomers can provide different LLDPE properties. Therefore, the use of long chain comonomers is also attractive for future production. 

In this work, the effects of short and long comonomer chain length on the polymerization activity and the properties of the resulting copolymers were investigated. The synthesis of the LLDPE was performed by copolymerization of ethylene and various 1-olefins, namely 1-hexene (1-C_6_), 1-octene (1-C_8_), 1-decene (1-C_10_), 1-dodecene (1-C_12_), 1-tetradecene (1-C_14_) and 1 -octadecene (1-C_18_), with a metallocene catalyst.

## 2. Results and Discussion

### 2.1. Homo- and co-polymerization activities

This study is aimed to investigate the polymerization of ethylene with short and long chain 1-olefins, namely 1-hexene, 1-octene, 1-decene, 1-dodecene, 1-tetradecene and 1-octadecene. The catalytic activities obtained with different 1-olefins are shown in Table 1.

**Table 1:** Copolymerization of ethylene with long chain 1-olefins using *rac*-Et[Ind]_2_ZrCl_2_/MAO, as the catalytic system.

Run number	Olefin type	Polymerization time (s)	Polymer yield^a^ (g)	Catalytic activity^b^ (×10^-4^kgPol/molZr h)
1	-	115	0.8703	1.8
2	1-C_6_	124	1.4781	2.9
3	1-C_8_	97	1.5529	3.8
4	1-C_10_	115	1.6783	3.5
5	1-C_12_	109	1.6134	3.6
6	1-C_14_	89	1.3704	3.7
7	1-C_18_	123	2.3157	4.5

^a^ The polymer yield was limited by the amount of ethylene fed (0.018 mol). The molar ratio of ethylene:comonomer was 2:1; ^b^ Activities were measured at polymerization temperature of 343 K, [ethylene]= 0.018 mol, [Al]_MMAO_ / [Zr]_cat_ = 1135, in toluene with total volume = 30 mL and [Zr]_cat_ = 5 × 10^-5^ M.

**Figure 1:**
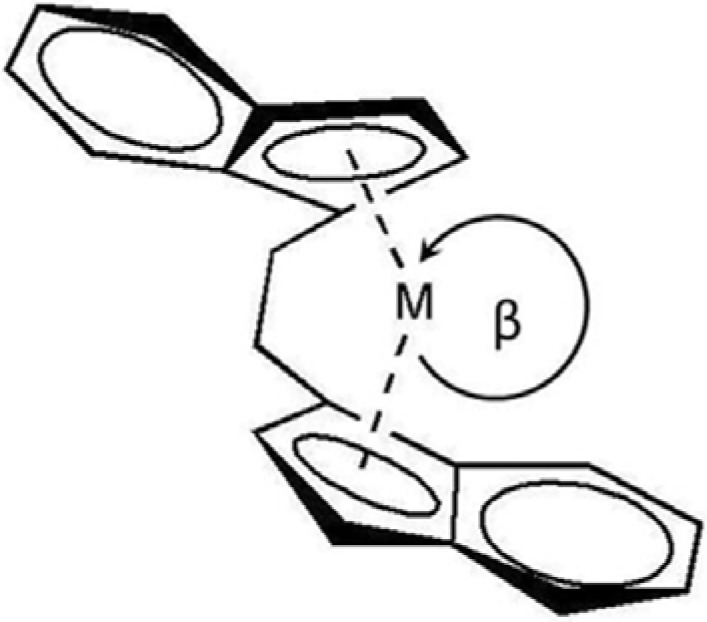
Structure of the opening gap aperture between C_p_(centroid)-M-C_p_-(centroid) in metallocene complex, redrawn from the conceptual idea by Braunschweig and Breitling [18].

From Table 1, when comparing the activities between homo-polymerization and co-polymerization, it can be seen that the addition of the comonomer in the system yields better activity. The enhancement of polymerization rate by 1-olefin comonomers, well documented for both titanocene [7,10,11] and zirconocene catalysts [7,12,13], is called the “comonomer effect”. This phenomenon in co-polymerization involving the zirconocene catalysts may be related to the perturbations of the ion pairs at the active sites. Karol *et al*. [13] have proposed that 1-olefins can function as ligands. By coordination to the active center, the 1-olefin can alter the charge density on the cationic zirconocenium ion. Metal centers with higher mobility, lower steric interference, and higher electrophilicity are believed to form stronger ion pairs. Monomers that cause a greater separation between the cationic metal centers and the MAO aggregates can enhance the activity of the catalyst, consequently increase the rate polymerization. On the other hand, two distinct cases can be described for the effect of the comonomer length. Considering the short chain length comonomers (runs 2-4), the results indicated that the increase of the comonomer length (from C_8_ to C_10_) resulted in lower activity due to increased steric hindrance. The longer chain comonomer can hinder the insertion of ethylene, and slow the propagation reaction process. This leads to lower catalytic activity for polymerization [12,13,14,15]. On the contrary, for the long chain length 1-olefins (runs 5-7), we observed an increase of polymerization activity when the length of 1-olefin was increased. This may be attributed to the opening of the gap aperture between C_p_(centroid)-M-C_p_-(centroid) in metallocene complex, which forced the coordination site to open more (Figure 1). This effect caused ethylene monomer to polymerize easier at the catalytic sites, and thus the activity increased [16,17]. A similar behavior was observed by Kaminsky *et al*. [17] for ethylene/ long chain 1-olefins copolymerization with a [Ph_2_C(2,7-di-*tert*-BuFlu)(Cp)]ZrCl_2_/MAO catalyst system, under different experimental conditions (*T* = 60 °C and the presence of hydrogen), but no reason was given for the trend.

The obtained result is also consistent with the study of Braunschweig and Breitling [18], which revealed that opening of the βC_p_(centroid)-M-C_p_-(centroid) angle can be found in the polymerization of ethylene and long chain olefins. Moreover, they also reported that a longer 1-olefin chain can open the C_p_(centroid)-M-C_p_-(centroid) angle wider in metallocene complexes. 

### 2.2. Polymer properties

Homo- and copolymers with different 1-olefins (copolymers containing 6 to 18 carbon atoms), synthesized by a metallocene catalyst, have been analyzed using four characterization techniques.

#### 2.2.1. SEM measurements

**Figure 2:**
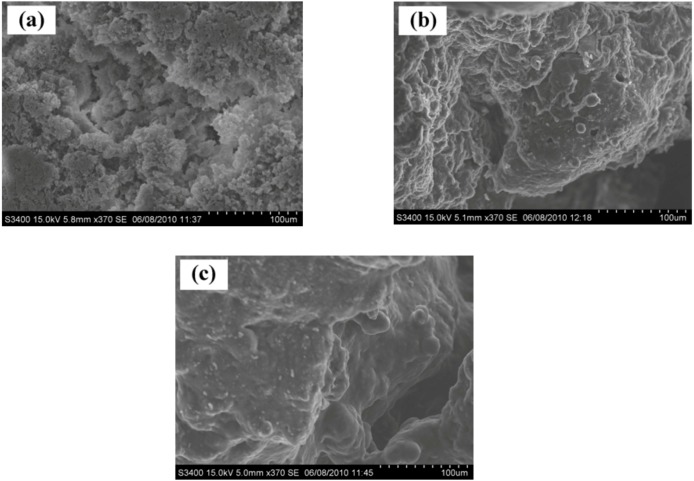
SEM micrograph of LLDPE produced with metallocene catalyst. **(a)** homopolymer **(b)** ethylene/1-hexene copolymer (**c)** ethylene/1-octadecene copolymer.

Figure 2 presents the scanning electron micrograph (SEM) of the polymers obtained by homo- and co-polymerization. Considering the effect of the length of comonomer on morphology, the results indicated that the crystalline structure of the obtained polymer seems to be lower with increased comonomer chain length. This is probably due to more steric hindrance caused upon introducing a longer chain length comonomer. Therefore, the amount and chain length of comonomer apparently affected on the morphology of the resulting polymer. 

#### 2.2.2. X-ray Diffraction (XRD) analysis

**Figure 3:**
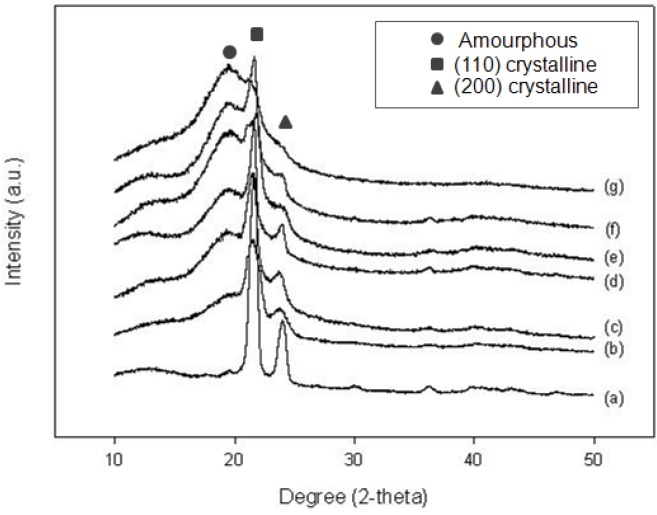
X-ray diffractograms of different samples. From bottom to top. **(a)** homopolymer **(b)** ethylene/1-C_6_ (**c)** ethylene/1-C_8_ **(d)** ethylene/1-C_10_ **(e)** ethylene/1-C_12_ **(f)** ethylene/1-C_14_ and **(g)** ethylene/1-C_18_ copolymers.

For XRD results, the diffractograms of the different samples, which were acquired at room temperature, are shown in **Figure 3**. As expected, it can be seen that all samples display XRD peaks at three positions. A broad amorphous peak was evident centered around 19.5-20 degrees. A previous work suggested this peak as indicative of the side branches of 1- olefin participating in the crystalline structure. While the other two peaks appeared at 2θ = 21.8 and 24.3 degrees are the (110) and (200) reflections, assigned to the characteristic orthorhombic cell of polyethylene [19,20,21]. Moreover, the longer chain length of the additional comonomers seemed to disturb the polymer recrystallization, which can probably be attributed to the increased steric hindrance, leading to a reduction in crystalline peak intensity, but clearly increasing the intensity of the amorphous peak [19,22].

#### 2.2.3. NMR analysis

**Figure 4:**
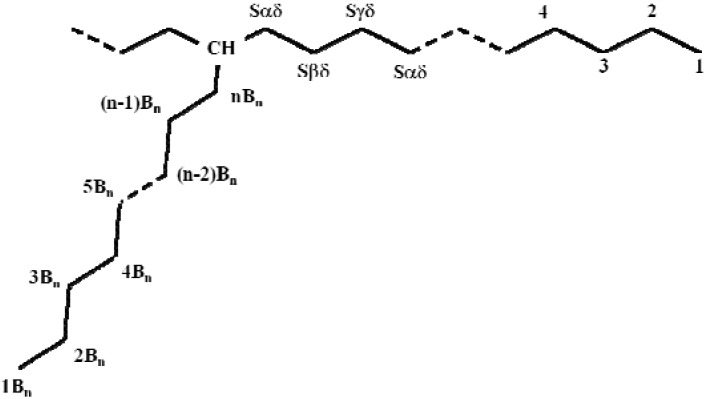
Resonance of the main chain methylenes and the side chain of the ethylene/1-olefin copolymer.

In order to determine the influence of chain length on the comonomer distribution, the obtained copolymers were also characterized by ^13^C-NMR measurements. The chemical-shift assignments of ethylene/ 1-C_12_ to ethylene/ 1-C_18_ copolymer and some resonances of the main and side chains are shown in Table 2 and Figure 4. The quantitative analysis of triad distribution for all copolymers is reported in Table 3. 

**Table 2:** Chemical-shift assignment in ^13^C-NMR spectra of ethylene/1-dodecene, ethylene/1-tetradecene and ethylene/1-octadecene copolymers [23].

Carbon type^a^	Chemical shift^b^ (ppm)		
	ethylene/1-dodecene (1-C_12_)	ethylene/1-tetradecene (1-C_14_)	ethylene/1-octadecene (1-C_18_)
1B_n_	14.10	14.10	14.10
2 B_n_	22.80	22.80	22.80
3 B_n_	32.13	32.13	32.13
4 B_n_	29.50	29.50	29.50
5 B_n_	29.85	29.85	29.85
6 B_n_	29.90^c^	29.90^ c^	29.90^ c^
7 B_n_	29.90^ c^	29.90^ c^	29.90^ c^
8 B_n_	30.36	29.90^ c^	29.90^ c^
9 B_n_	27.18	29.90^ c^	29.90^ c^
10 B_n_	34.45	30.37	29.90^ c^
11 B_n_	-	27.18	29.90^ c^
12 B_n_	-	34.46	29.90^ c^
13 B_n_	-	-	29.90^ c^
14 B_n_	-	-	30.37
15 B_n_	-	-	27.18
16 B_n_	-	-	34.46
CH	38.11	38.11	38.11
Sαδ	34.49	34.49	34.49
Sβδ	27.20	27.20	27.20
Sγδ	30.38	30.38	30.38
Sδδ	29.90	29.90	29.90

^a^ See Scheme 1; ^b^ According to Randall [26]; ^c^ Overlapped to S δδ peak at 29.90 ppm.

**Table 3:** Triad distribution and properties of the resulting ethylene/1-olefin copolymers.

Entry	Olefin type	Triad distribution^a^						C_n_ (mol%)^b^	T_m_ (^o^C)^c^	*χ_c_* (%)^d^	d^e^ (g/mL)
		[EEE]	[XEE]	[EXE]	[XEX]	[EXX]	[XXX]				
2	1-C_6_	0.775	0.142	0.052	0.017	0.014	0.000	7	121.64	7.36	0.88
3	1-C_8_	0.412	0.379	0.164	0.045	0.000	0.000	16	113.62	2.44	0.88
4	1-C_10_	0.471	0.310	0.176	0.043	0.000	0.000	18	116.25	2.33	0.88
5	1-C_12_	0.499	0.331	0.135	0.035	0.000	0.000	13	117.61	6.21	0.89
6	1-C_14_	0.604	0.154	0.091	0.087	0.064	0.000	20	112.98	1.33	0.87
7	1-C_18_	0.591	0.214	0.152	0.043	0.000	0.000	15	117.58	4.34	0.88

^a^ Obtained from ^13^C-NMR, where E refers to ethylene monomer and X refers to 1-olefin comonomer; ^b^ Content of 1-olefin in the copolymer from ^13^C-NMR; ^c^ Melting temperature from DSC; ^d^ Crystallinity degree: *χ_c_* =100 × (ΔH/ΔH°) , where ΔH° = 290 J/g for linear polyethylene; ^e^ Copolymer density calculated from the semi-empirical equation: d = (2,195 + ΔH)/2,500.

From the table, it is found that ethylene incorporation in all systems gave copolymers with similar triad distribution, and only random copolymers can be produced in all systems. However, the olefin length has no effect on the comonomer distribution [12,23,24,25]. It should be noted that the comonomer content depends on different variables; for example, a long 1-olefin chain length forces the angle of the metallocene complex to open more. However, at the same time it causes steric hindrance to the incoming ethylene monomer. Therefore, the two effects (chain length and opened angle) can be superimposed on each other.

#### 2.2.4. Differential scanning calorimetric analysis

When dealing with the thermal properties of the polymer, DSC measurements are usually considered the second melting of the sample. All the experimental results, including melting temperature (T_m_), % crystallinity (χ_c_), and density are also reported in Table 3. The PE sample (run 1) is a high density polymer with a linear microstructure, a high melting temperature (Tm~135 °C), a degree of crystallinity of 65% and a high density (0.95 g/mL) (data not shown in the Table). Based on Table 3, it can be observed that the polymer with higher incorporation of comonomers exhibited less crystallinity and lower melting temperature. This is in correspondence with the percent insertion from ^13^C-NMR results. In addition, when the crystallinity results obtained from DSC measurements are considered, they indicate that the length of the comonomer did not affect the crystallization behavior, which was different from the XRD results. This was due to the fact that XRD was analyzed the assyn-polymers at ambient conditions, whereas the DSC measurement was performed upon heating the samples. For DSC, the determination relies on the measurement of the enthalpy of melting and on the assumption of a unique enthalpy of melting for the crystal. Thus, the enthalpy of melting of long 1-olefin side chains had to be taken into account for the determination of crystallinity. Therefore, the crystallinity value may be different between the various determinations. [12,13,27,28,29]. However, it can be concluded that the increase in the length of the comonomer chain can result in a decrease of the melting temperature. The density of all samples is in the range of 0.87-0.95 g/cm^3^ indicating a typical LLDPE structure. 

## 3. Experimental

### 3.1. Materials

Chemicals and polymerizations were handled and operated under an argon atmosphere, using a glove box and/or Schlenk techniques. Toluene was dried over dehydrated CaCl_2_, and then distilled over sodium/benzophenone before use. The zirconocne, (*rac*-Et[Ind]_2_ZrCl_2_) was supplied by the Aldrich Chemical Company, Inc. Modified methylaluminoxane (MMAO) in hexane was donated by Tosoh (Akso, Japan). 1-Olefins were purchased from Aldrich Chemical Company, Inc. Ethylene gas (99.96% pure) was donated by the National Petrochemical Co., Ltd., Thailand. Ultrahigh purity argon was further purified by passing it through columns packed with BASF R3-11G catalyst (molecular-sieved to 3 Å), sodium hydroxide (NaOH), and phosphorus pentaoxide (P_2_O_5_) to remove traces of oxygen and moisture. 

### 3.2. Homo- and co-polymerization

Ethylene/1-olefin copolymerizations was carried out in a 100 mL semi-batch stainless steel autoclave reactor equipped with a magnetic stirrer. In the glove box, the desired amounts of *rac*-Et[Ind]_2_ZrCl_2_ and MMAO were introduced into the autoclave and then, toluene was added (to make a total volume of 30 mL). After that, the reactor was frozen in liquid nitrogen to stop any reactions and the proper amount of the comonomer was injected into the reactor (the molar ratio of ethylene:comonomer was fixed at 2:1). The reactor was evacuated to remove argon. Then, it was heated up to polymerization temperature (343 K) and the polymerization was started by feeding ethylene gas until the consumption of 0.018 mol of ethylene (6 psi was observed from the pressure gauge) was reached. The polymerization was terminated by addition of acidic methanol [30]. The time of reaction was recorded for purpose of calculating the activity. The precipitated polymer was washed with acidic methanol and dried at room temperature. Based on the system as mentioned above, the polymer yield was fixed by the amount of ethylene fed (0.018 mol). Experimentally, the polymerization was performed at least three times for each run and only the average yield and activity are reported. The error was found to be within less than 5% based on this polymerization system.

### 3.3. Polymer characterization

Scanning electron microscopy (SEM) was used to determine the morphology of the polymeric samples. The samples were sputter-coated with a fine layer of platinum in an Edward Sputter Coater and analyzed with a JEOL (mode JSM-6400) electron microscope.

X-ray diffraction (XRD) was performed to determine the bulk crystalline phases of samples. Diffraction patterns were recorded in the reflection mode at room temperature using a Siemens D-5000 instrument. Ni-filtered Cu K_α_ (λ = 1.54439 Å) was used. The diffraction scans were collected over a period of 2.4° min^-^1 of 2θ from 10 to 80°.

^13^C-NMR spectroscopy was used to determine the triad distribution and 1-olefin insertion indicating the copolymer microstructure. Chemical shifts were referenced internally to the CDCl_3_ peak and calculated according to the method described by Randall [26]. Sample solutions were prepared by dissolving copolymer (50 mg) in 1,2,4-trichlorobenzene and CDCl_3_ (0.5 mL). ^13^C-NMR spectra were taken at 383 K using a Bruker Avance II 400 operating at 100 MHz with an acquisition time of 1.5 s and a delay time of 4 s.

The thermal properties were measured by a PerkineElmer Pyris Diamond Differential Scanning Calorimeter at a standard heating/cooling rate of 20K/min, under nitrogen flow. Both first and second melting temperatures have been analyzed. The reported melting temperature values are referred to the second heating scan. The peak temperature was assumed as melting temperature (T_m_) and the area was corresponding to the global melting enthalpy (ΔH). The crystallinity, *χ_c_*, was calculated from DSC data by using the formula *χ_c_* =100·× (ΔH/ΔH°) where ΔH° = 290 J/g is the enthalpy of fusion for linear polyethylene as reported in ref. [31]. Approximately, 5-10 mg of sample was used for each DSC measurement. Moreover, it was possible to relate ΔH (J/g) to the density (d, g/mL) of the copolymer through the following semiempirical equation: d = (2,195 + ΔH)/2,500. 

## 4. Conclusions

In this article, we have reported the synthesis of LLDPE from the copolymerization of ethylene/1-olefin via metallocene/MAO catalysts by varying the comonomer (1-olefin) chain length. The results show that the increase in the short chain length 1-olefns (from 1-C_8_ to 1-C_10_) can cause more steric hindrance of the catalytic center leading to decreased activity. However, when a long chain comonomer was used, a block of long chain 1-olefin can force the opening of the supplementary angle, therefore the coordination site was more open, resulting in increased activity. However, the increase of the chain length has no significant effects on the melting temperature and comonomer distribution. The crystallinity tended to decrease with increased chain length of comonomer, based on the XRD measurements.
